# The Association of Inflammatory Markers With Nonalcoholic Fatty Liver Disease Differs by Human Immunodeficiency Virus Serostatus

**DOI:** 10.1093/ofid/ofx153

**Published:** 2017-07-23

**Authors:** Jennifer C Price, Ruibin Wang, Eric C Seaberg, Matthew J Budoff, Lawrence A Kingsley, Frank J Palella, Mallory D Witt, Wendy S Post, Chloe L Thio

**Affiliations:** 1 Division of Gastroenterology and Hepatology, Department of Medicine, University of California San Francisco; 2 Department of Epidemiology, Johns Hopkins Bloomberg School of Public Health, Baltimore, Maryland; Divisions of; 3 Cardiology and; 4 HIV Medicine, Department of Medicine, Los Angeles Biomedical Research Institute at Harbor-UCLA Medical Center, Torrance, California; 5 Departments of Infectious Diseases and Microbiology and Epidemiology, University of Pittsburgh Graduate School of Public Health, Pennsylvania; 6 Division of Infectious Diseases, Department of Medicine, Northwestern University Feinberg School of Medicine, Chicago, Illinois; Divisions of; 7 Cardiology and; 8 Infectious Diseases, Department of Medicine, Johns Hopkins University School of Medicine, Baltimore, Maryland

**Keywords:** HIV, fatty liver, NAFLD, biomarkers, adiponectin

## Abstract

**Background:**

We aimed to determine the relationship of circulating adipokines and inflammatory biomarkers with fatty liver among men in the Multicenter AIDS Cohort Study.

**Methods:**

Noncontrast computed tomography was used to assess fatty liver and measure abdominal visceral adipose tissue (VAT) area in 526 participants without history of cardiovascular disease, heavy alcohol use, or viral hepatitis infection. Multivariable logistic regression was used to assess associations of circulating biomarker levels with fatty liver.

**Results:**

Three hundred twenty-nine human immunodeficiency virus (HIV)-infected men had higher levels of several inflammatory biomarkers compared with 197 HIV-uninfected men. Among HIV-uninfected men, increased adiponectin was associated with lower odds of fatty liver (odds ratio [OR] = 0.51 per doubling, *P* = .02), whereas higher odds of fatty liver was observed with increased levels of the proinflammatory markers intercellular adhesion molecule (ICAM)-1 (OR = 5.30, *P* = .004), C-reactive protein (OR = 1.66, *P* = .002), interleukin (IL)-6 (OR = 1.67, *P* = .03), and tumor necrosis factor α receptor 2 (OR = 6.55, *P* = .003). Among HIV-infected men, ICAM-1 was the only proinflammatory marker associated with greater odds of fatty liver (OR = 2.67, *P* = .02), whereas higher adiponectin (OR = 0.57, *P* = .003), and osteoprotegerin levels (OR = 0.48, *P* = .03) were associated with lower odds. These associations were all independent of VAT.

**Conclusions:**

Fatty liver is associated with a heightened inflammatory state independent of visceral adiposity in HIV-uninfected men but not in HIV-infected men. However, a heightened anti-inflammatory state may protect against fatty liver regardless of HIV serostatus.

Nonalcoholic fatty liver disease is a leading cause of liver disease in the United States and among human immunodeficiency virus (HIV)-infected persons, affecting approximately 30% of both the general and HIV-infected populations without chronic viral hepatitis infection [1, 2]. Elevations in levels of various circulating inflammatory biomarkers have been associated with fatty liver among HIV-uninfected individuals, but little is known about these markers in the setting of HIV infection [3]. Although chronic immune activation is a hallmark of HIV infection [4], in a prior study we unexpectedly found that HIV infection was not associated with increased odds of fatty liver among participants in the Multicenter AIDS Cohort Study (MACS) [5]. This finding has been confirmed in other cohorts [2, 6] and raises questions about the relationship between inflammation and fatty liver among HIV-infected individuals.

Most studies evaluating the association of inflammatory biomarker levels and fatty liver lack direct measurements of visceral adipose tissue (VAT), a well established independent risk factor for fatty liver disease [5–7]. Adipose tissue secretes a variety of biologically active factors, including the anti-inflammatory adipokine adiponectin, which is reduced in obesity, and proinflammatory cytokines such as tumor necrosis factor (TNF)α and interleukin (IL)-6, which are increased in obesity. Reductions in adiponectin and elevations in TNFα and IL-6 have been linked to fatty liver development [3]. Although these biomarkers appear to mediate the causal pathway between VAT and fatty liver, it is unclear whether they are also associated with fatty liver independent of VAT. This is important because fatty liver may be an independent risk factor for cardiovascular disease, and heightened chronic systemic inflammation is a postulated mechanism underlying this association [8]. Indeed, lower circulating levels of adiponectin and higher levels of IL-6 and soluble TNFα receptor (TNFαR)2 are associated with subclinical cardiovascular disease among HIV-infected men in the MACS [9–11].

The aim of this study was to evaluate associations of adipokines and inflammatory biomarkers with fatty liver in HIV-infected and HIV-uninfected men in the MACS. We hypothesized that (1) adiponectin and inflammatory biomarkers would be associated with fatty liver disease independent of VAT and (2) the relationship of these markers with fatty liver disease would differ by HIV serostatus.

## METHODS

### Study Design and Participants

We performed a cross-sectional study within the MACS, an ongoing prospective cohort study of men who have sex with men. Details of study recruitment and participant characteristics have been described elsewhere [12, 13]. Men were recruited from 4 sites in the United States (Baltimore, MD/Washington DC; Chicago, IL; Pittsburgh, PA; and Los Angeles, CA) and were followed semiannually for interview, physical examination, and laboratory testing. From January 2010 to August 2013, 1006 men were enrolled in the MACS cardiovascular disease substudy and underwent computed tomography (CT) imaging [14]. Exclusion criteria for the substudy were weight >300 pounds, history of cardiac surgery, or history of coronary angioplasty or stent placement. Among the substudy participants, 829 had adequate visualization of the liver and spleen on noncontrast cardiac CT. After excluding men who consumed 3 or more alcoholic drinks per day, were infected with hepatitis C or hepatitis B virus, or were missing key covariate data including adipokine or biomarker level testing, 526 men were included in the final fatty liver and biomarker level analysis (Supplemental Figure). The study was approved by the Institutional Review Board at each site, and all participants signed informed consent.

### Fatty Liver and Adipose Tissue Measurements

Multidetector row CT scanning was performed on each participant, and each scan was reviewed by a single reader who was blinded to all demographic and clinical data. Fatty liver was defined as a liver attenuation/spleen attenuation ratio <1.0 on noncontrast CT, as previously described [5]. Visceral adipose tissue and subcutaneous adipose tissue (SAT) areas were measured from a single CT slice in the space between the fourth and fifth lumbar vertebrae [15].

### Biomarker Measurements

As part of the MACS cardiovascular disease substudy, biomarkers were selected a priori as possible predictors of cardiovascular disease and were measured from blood samples collected at the time of the CT scanning. The specific biomarkers selected and their biologic activities were as follows: adiponectin (anti-inflammatory adipokine), leptin (proinflammatory adipokine), high-sensitivity C-reactive protein ([CRP] acute phase reactant), D-dimer (acute phase reactant), fibrinogen (acute phase reactant), cystatin C (marker of renal function), soluble (s)CD14 (monocyte activation), sCD163 (macrophage activation), monocyte chemoattractant protein 1 ([MCP-1] monocyte migration), intercellular adhesion molecule 1 ([ICAM-1] endothelial cell activation), IL-6 (systemic inflammation), IL-18 (systemic inflammation), receptor activator of nuclear factor κB ligand ([RANKL] TNF cytokine family), osteoprotegerin (member of TNF receptor superfamily), TNFαR1 (TNFα receptor), and TNFαR2 (TNFα receptor).

Interleukin-6 (serum) levels were measured using chemiluminescent enzyme-linked immunosorbent assay ([ELISA] R&D Systems, Minneapolis, MN). Adiponectin (serum), leptin (serum), sCD14 (plasma), sCD163 (serum), and ICAM-1 (serum) levels were also measured using ELISA. Fibrinogen (plasma), CRP (serum), and cystatin C (plasma) levels were measured using the Siemens BNII Nephelometer (Siemens Healthcare Diagnostics, Deerfield, IL). D-dimer (plasma) levels were measured using a Stago STA-R analyzer (Parsippany, NJ). Monocyte chemoattractant protein 1 (plasma) was measured using a singleplex cytokine panel (Millipore, Billerica, MA); sTNFαR1 (plasma) and sTNFαR2 (plasma) levels were measured using a Milliplex soluble cytokine receptor panel (Millipore, Billerica, MA); RANKL (plasma) was measured using a singleplex bone kit (Millipore, Billerica, MA); and osteoprotegerin (serum) was measured using ELISA (Alpco Diagnostics, Salem, NH). Interleukin-18 (serum) was measured using ELISA (Platinum ELISA; eBiosciences, San Diego, CA). The interassay coefficient of variation ranges for the biomarker levels are listed in the Supplementary Methods.

### Demographic and Clinical Characteristics

Additional participant level covariates were obtained from the most proximal MACS visit before the CT scan, which was a median of 2 months before the CT visit (interquartile range, 1–4 months). Age, race, alcohol use, and medication use were obtained by self-report. Body mass index (BMI) was calculated as body weight (kg)/[height (m)]^2^. Genotyping of *PNPLA3* (rs738049) was performed using TaqMan SNP genotyping assays (Life Technologies, Carlsbad, CA). Hypertension was defined as systolic blood pressure (BP) >140 mmHg, diastolic BP >90 mmHg, or self-reported use of an antihypertensive medication. Fasting insulin, glucose, triglycerides, alanine aminotransferase (ALT), aspartate aminotransferase (AST), CD4^+^ T-cell count, and plasma HIV ribonucleic acid (RNA) levels were measured from blood collected at the last MACS visit before CT scan, generally within 6 months. Diabetes was defined as a fasting glucose ≥126 mg/dL or self-reported use of a medication to treat diabetes. The homeostatic model assessment of insulin resistance (HOMA-IR) was calculated using fasting insulin, and glucose values and was dichotomized as <4.9 or ≥4.9 based on our prior analysis [5].

### Statistical Analysis

We compared demographic and other cofactors of interest by HIV serostatus and presence or absence of fatty liver using the Wilcoxon rank-sum test for continuous variables and χ^2^ test for categorical variables.

To better understand the relationships between the biomarker levels, VAT, and fatty liver in our cohort, we performed linear regression to determine which biomarkers were associated with VAT on univariate analysis and on multivariable analysis adjusting for MACS site, age, race, and HIV serostatus. We also evaluated the correlation between the biomarker levels and HIV-specific factors, including current and nadir CD4^+^ T-cell count, current plasma HIV RNA levels, current and cumulative highly active antiretroviral therapy (HAART) use, cumulative lamivudine use, and cumulative dideoxynucleoside use (defined as didanosine, zidovudine, stavudine, or zalcitabine).

Next, we constructed logistic regression models in a sequential fashion with fatty liver as the outcome and the biomarker levels as the primary predictors. Our primary multivariable analyses adjusted for MACS site, age, race, and *PNPLA3* genotype (Model A) and Model A + VAT. We also performed a sensitivity analysis including Model A + HOMA-IR and Model A + HOMA-IR + VAT. Among the HIV-infected men, we performed a separate analysis adjusting for HIV-specific variables including current CD4 count, HIV RNA <50 copies/mL, cumulative HAART use, and cumulative dideoxynucleoside use. For both the linear and logistic regression models, all biomarker levels were log base 2-transformed. Thus, the coefficients of the models represent the difference in VAT or the odds ratio (OR) of fatty liver per doubling of the biomarker level. Each biomarker was treated as an independent exposure and evaluated separately in the multivariable regression models. Potential interactions between HIV serostatus and individual biomarkers were assessed using the likelihood ratio test. Finally, we performed a sensitivity analysis excluding HIV-infected men with detectable viral load (HIV RNA ≥50 copies/mL, N = 59). Statistical analyses were performed using Stata/SE version 13.1 (StataCorp, College Station, TX) and R version 3.3.2 (The R Foundation for Statistical Computing, Vienna, Austria).

## RESULTS

### Study Population

Among the 329 HIV-infected and 197 HIV-uninfected men, HIV-infected men were slightly younger and were more likely to be non-Hispanic white (Table 1). The median VAT area was similar between the HIV-infected and -uninfected men despite lower median SAT area and slightly lower median BMI in the HIV-infected men. The majority of the HIV-infected men (88%) was on HAART, and most had undetectable HIV RNA levels (82%). Significant differences in biomarker levels between the HIV-infected and -uninfected groups included lower levels of RANKL and leptin but higher levels of inflammatory biomarkers CRP, IL-18, TNFαR2, sCD14, sCD163, ICAM-1, MCP-1, cystatin C, and osteoprotegerin in HIV-infected men (Table 2).

**Table 1.** Characteristics of the Study Population by HIV and Fatty Liver Status^a^

Characteristics	HIV^+^ (N = 329)	HIV^−^ (N = 197)	Fatty Liver (N = 80)	No Fatty Liver (N = 446)
Demographics				
Race, N (%)				
White non-Hispanic	**182 (55**)	**131 (66**)	**57 (71**)	**256 (57**)
Black non-Hispanic	**108 (33**)	**47 (24**)	**12 (15**)	**143 (32**)
Other	**39 (12**)	**19 (10**)	**11 (14**)	**47 (11**)
Age (years), median (IQR)	**52 (47–57**)	**54 (50–62**)	54 (48–59)	53 (48–59)
Comorbidities				
BMI (kg/m^2^), median (IQR)	**26 (23–29**)	**27 (24–30**)	**28 (26–32**)	**25 (24–29**)
Abdominal VAT (mm^2^), median (IQR)	144 (87–212)	147 (92–210)	**217 (136–314**)	**137 (81–197**)
Abdominal SAT (mm^2^), median (IQR)	**173 (112–262**)	**227 (163–299**)	**231 (161–339**)	**188 (122–269**)
Diabetes, N (%)	39 (12)	17 (9)	13 (17)	43 (10)
HOMA-IR ≥4.9, N (%)	82 (25)	36 (18)	**40 (50**)	**78 (17**)
On lipid-lowering agent, N (%)	120 (37)	59 (31)	29 (37)	150 (34)
Triglycerides (mg/dL), median (IQR)	**130 (91–205**)	**107 (77–147**)	**148 (109–217**)	**116 (82–172**)
Hypertension, N (%)	152 (48)	85 (45)	**45 (59**)	**192 (44**)
ALT (U/L), median (IQR)	**25 (18–35**)	**21 (17–28**)	**30 (21–43**)	**22 (17–30**)
AST (U/L), median (IQR)	**24 (20–31**)	**21 (18–25**)	**24 (21–32**)	**22 (18–27**)
FIB-4 3.25	**9 (3**)	**0 (0**)	0 (0)	9 (2)
*PNPLA3 (rs738409*), N (%)				
CC	201 (61)	115 (58)	**35 (44**)	**281 (63**)
GC	112 (34)	72 (37)	**41 (51**)	**143 (32**)
GG	16 (5)	10 (5)	**4 (5**)	**22 (5**)
HIV-related characteristics				
HIV-infected, N (%)			44 (55)	285 (64)
Undetectable viral load (<50 copies/mL), N (%)	270 (82)		39 (89)	231 (81)
Current CD4 cell count (cells/mm^3^), median (IQR)	598 (438–776)		627 (497–853)	593 (422–760)
Current HAART, N (%)	291 (88)		41 (93)	250 (88)
Cumulative HAART (years), median (IQR)	9 (6–12)		10 (8–13)	9 (6–12)
Cumulative dideoxy use (years), median (IQR)	8 (3–12)		**10 (5–13**)	**7 (3–12**)

Abbreviations: ALT, alanine aminotransferase; AST, aspartate aminotransferase; BMI, body mass index; FIB-4, fibrosis-4 index; HAART, highly active antiretroviral therapy; HIV, human immunodeficiency virus; HOMA-IR, homeostatic model assessment of insulin resistance; IQR, interquartile range; SAT, subcutaneous fat area; VAT, visceral adipose tissue.

^a^Bold signifies statistical significance (*P* < .05). Comparisons were between HIV positive and HIV negative and between fatty liver and no fatty liver.

**Table 2.** Biomarker Levels by HIV Serostatus^a^

Biomarker	HIV^+^ (N = 329)	HIV^−^ (N = 197)	*P* Value
sCD14 (ng/mL), median (IQR)	**1597 (1389–1841**)	**1294 (1135–1471**)	**<.001**
sCD163 (ng/mL), median (IQR)	**642 (486–822**)	**535 (447–674**)	**<.001**
Adiponectin (ng/mL), median (IQR)	6167 (3955–9896)	7038 (4916–9539)	.16
CRP (µg/mL), median (IQR)	**1.3 (0.7–2.9**)	**1.1 (0.6–2.1**)	**.03**
Cystatin C (mg/L), median (IQR)	**0.8 (0.8–1.0**)	**0.8 (0.7–0.9**)	**<.001**
D-dimer (µg/mL), median (IQR)	0.2 (0.1–0.3)	0.2 (0.1–0.3)	.14
Fibrinogen (mL/dL), median (IQR)	327 (279–376)	332 (301–377)	.06
ICAM-1 (ng/mL), median (IQR)	**251 (214–298**)	**224 (197–268**)	**<.001**
IL-6 (pg/mL), median (IQR)	1.4 (1.0–2.2)	1.3 (0.9–2.0)	.11
IL-18 (pg/mL), median (IQR)	**309 (211–448**)	**227 (163–354**)	**<.001**
Leptin (pg/mL), median (IQR)	**5208 (2260–9828**)	**6384 (3547–11 320**)	**.01**
MCP1 (pg/mL), median (IQR)	**268 (208–350**)	**234 (184–299**)	**<.001**
Osteoprotegerin (pmol/L), median (IQR)	**4.6 (3.9–5.6**)	**4.4 (3.6–5.2**)	**.02**
RANKL (pg/mL), median (IQR)	**8 (2–21**)	**14 (8–28**)	**<.001**
TNFαR1 (pg/mL), median (IQR)	1137 (964–1425)	1156 (979–1377)	.89
TNFαR2 (pg/mL), median (IQR)	**6343 (5346–7628**)	**5834 (5077–6852**)	**.002**

Abbreviations: CRP, C-reactive protein; HIV, human immunodeficiency virus; ICAM, intercellular adhesion molecule; IL, interleukin; IQR, interquartile range; MCP, monocyte chemoattractant protein; RANKL, receptor activator of nuclear factor κB ligand; s, soluble; TNFαR, tumor necrosis factor α receptor.

^a^Bold signifies statistical significance (*P* < .05).

Fatty liver was identified in 80 individuals (15%): 44 HIV-infected (13%) and 36 HIV-uninfected (18%). Compared with men without fatty liver, those with fatty liver were more likely to be non-Hispanic white and have *PNPLA3* non-CC genotype and had higher median BMI, greater abdominal VAT and SAT, higher HOMA-IR scores, and higher triglyceride levels (Table 1). Among HIV-infected men, those with fatty liver had longer cumulative dideoxynucleoside exposure. Detailed analysis of demographic and clinical factors associated with fatty liver in our cohort, including our finding of less fatty liver in the HIV-infected group, was previously published [5].

### Association of Serum Biomarkers With Visceral Adipose Tissue

Multivariable analysis involving the entire cohort revealed that lower adiponectin and higher CRP, IL-6, sCD163, cystatin C, TNFαR1, and leptin levels were each significantly associated with higher VAT volume after adjusting for MACS site, age, race, and HIV serostatus. Among HIV-uninfected men, the association of these biomarkers with VAT persisted, with higher ICAM-1 levels also independently associated with increased VAT (Supplemental Table 1). By contrast, among HIV-infected men, higher CRP, higher leptin, and lower adiponectin were associated with increased VAT on multivariable analysis.

### Association of Serum Biomarkers With Fatty Liver

**Figure 1.**
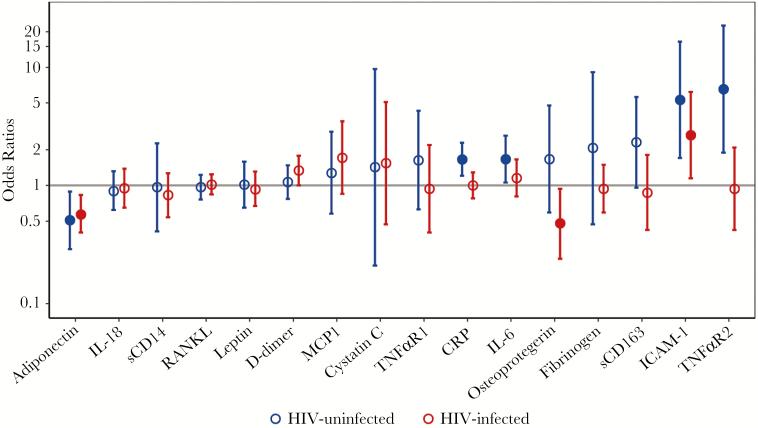
Odds ratios of fatty liver disease by human immunodeficiency virus (HIV) serostatus, presented on log_10_ scale. Biomarkers are modeled separately using multivariable logistic regression models. Odds ratios are calculated for each 2 times increase in the levels of biomarkers. All models were adjusted for Multicenter AIDS Cohort Study (MACS) study site, age, race, *PNPLA3* non-CC genotype, and area of abdominal visceral adipose tissue. Error bars represent 95% confidence intervals. Solid circles denote statistically significant odds ratios. ICAM, intercellular adhesion molecule; IL, interleukin; MCP, monocyte chemoattractant protein; RANKL, receptor activator of nuclear factor κB ligand; sCD14, soluble CD14; TNFαR, tumor necrosis factor α receptor.

Among HIV-uninfected men, after adjustment for age, race, MACS site, and *PNPLA3* genotype, we found a greater odds of fatty liver with higher levels of ICAM-1 (OR = 8.18, *P* < .001), CRP (OR = 2.02, *P* < .001), IL-6 (OR = 1.67, *P* = .004), TNFαR2 (OR = 9.11, *P* < .001), sCD163 (OR = 2.99, *P* = .01), and leptin (OR = 3.10, *P* = .002), and a lower odds of fatty liver with higher adiponectin levels (OR = 0.45, *P* = .002) (Figure 1, Table 3). To determine whether these associations were mediated by VAT, we added VAT as a covariate in the model and found that the associations with fatty liver were attenuated but persisted for ICAM-1 (OR = 5.30, *P* = .004), CRP (OR = 1.66, *P* = .002), IL-6 (OR = 1.67, *P* = .03), TNFαR2 (OR = 6.55, *P* = .003), and adiponectin (OR = 0.51, *P* = .02), whereas the relationships of sCD163 and leptin levels with fatty liver were no longer statistically significant.

**Table 3.** Association of Biomarkers with Fatty Liver by HIV Serostatus^a^

Biomarker	Multivariable model	HIV^+^ (N = 329)		HIV^−^ (N = 197)		*P* for Interaction
		Odds Ratio for Fatty Liver (95% CI)^b^	*P* Value	Odds Ratio for Fatty Liver (95% CI)^b^	*P* Value	
sCD14	Model A	0.72 (0.49–1.07)	.10	0.96 (0.46–1.99)	.90	.50
	+VAT	0.83 (0.54–1.27)	.40	0.97 (0.41–2.27)	.94	.80
sCD163	Model A	1.13 (0.59–2.18)	.72	**2.99 (1.30–6.90**)	**.01**	.06
	+VAT	0.87 (0.42–1.82)	.71	2.32 (0.96–5.61)	.06	.07
Fibrinogen	Model A	0.94 (0.58–1.51)	.79	2.81 (0.64–12.27)	.17	.15
	+VAT	0.94 (0.59–1.50)	.81	2.08 (0.47–9.08)	.33	.27
TNFαR2	Model A	1.19 (0.57–2.46)	.64	**9.11 (2.78–29.80**)	**<.001**	**.004**
	+VAT	0.94 (0.42–2.09)	.87	**6.55 (1.90–22.53**)	**.003**	**.01**
Adiponectin	Model A	**0.53 (0.38–0.74**)	**<.001**	**0.45 (0.27–0.74**)	**.002**	.71
	+VAT	**0.57 (0.40–0.83**)	**.003**	**0.51 (0.29–0.89**)	**.02**	.90
CRP	Model A	1.18 (0.94–1.49)	.15	**2.02 (1.49–2.74**)	**<.001**	**.01**
	+VAT	1.00 (0.78–1.29)	.99	**1.66 (1.21–2.30**)	**.002**	**.03**
Cystatin C	Model A	2.28 (0.81–6.40)	.12	4.59 (0.76–27.52)	.10	.41
	+VAT	1.55 (0.47–5.09)	.47	1.43 (0.21–9.66)	.72	.67
D-dimer	Model A	**1.32 (1.01–1.73**)	**.046**	1.19 (0.88–1.62)	.26	.56
	+VAT	1.34 (1.00–1.79)	.05	1.07 (0.77–1.48)	.69	.28
ICAM-1	Model A	**3.21 (1.45–7.12**)	**.004**	**8.18 (2.74–24.41**)	**<.001**	.09
	+VAT	**2.67 (1.15–6.19**)	**.02**	**5.30 (1.71–16.46**)	**.004**	.22
IL-18	Model A	1.03 (0.72–1.46)	.89	0.96 (0.70–1.31)	.79	.75
	+VAT	0.95 (0.65–1.39)	.79	0.90 (0.62–1.32)	.60	.87
IL-6	Model A	1.26 (0.92–1.72)	.14	**2.11 (1.38–3.24**)	**.001**	.06
	+VAT	1.16 (0.81–1.66)	.42	**1.67 (1.06–2.64**)	**.03**	.19
Leptin	Model A	**1.46 (1.12–1.91**)	**.005**	**1.67 (1.18–2.38**)	**.004**	.48
	+VAT	0.93 (0.67–1.31)	.70	1.02 (0.65–1.59)	.94	.58
MCP1	Model A	1.87 (0.96–3.65)	.07	1.32 (0.66–2.67)	.43	.35
	+VAT	1.72 (0.85–3.49)	.13	1.28 (0.58–2.85)	.54	.57
Osteoprotegerin	Model A	**0.52 (0.27–0.99**)	**.046**	1.73 (0.63–4.77)	.29	.08
	+VAT	**0.48 (0.24–0.94**)	**.03**	1.67 (0.59–4.76)	.34	.07
RANKL	Model A	0.99 (0.82–1.19)	.90	0.99 (0.79–1.24)	.94	.86
	+VAT	1.02 (0.84–1.24)	.82	0.97 (0.76–1.23)	.80	.82
TNFαR1	Model A	1.25 (0.58–2.74)	.57	2.34 (0.91–6.02)	.08	.18
	+VAT	0.94 (0.40–2.20)	.89	1.64 (0.63–4.29)	.32	.17

Abbreviations: CI, confidence interval; HIV, human immunodeficiency virus; ICAM, intercellular adhesion molecule; IL, interleukin; MACS, Multicenter AIDS Cohort Study; MCP, monocyte chemoattractant protein; OR, odds ratio; RANKL, receptor activator of nuclear factor κB ligand; s, soluble; TNFαR, tumor necrosis factor α receptor; VAT, abdominal visceral adipose tissue.

^a^Bold signifies statistical significance at *P* < .05.

^b^Odds ratios are calculated for each 2 times increase in the levels of biomarkers. Model A was adjusted for MACS study site, age, race, and *PNPLA3* genotype. *P* for interaction tested potential interaction between biomarkers and HIV serostatus.

Among HIV-infected men, after adjusting for age, race, MACS site, and *PNPLA3* genotype, there was a greater odds of fatty liver with increasing ICAM-1 (OR = 3.21, *P* = .004), D-dimer (OR = 1.32, *P* = .046), and leptin (OR = 1.46, *P* = .005), whereas a lower odds of fatty liver was associated with increasing adiponectin (OR = 0.53, *P* < .001) and osteoprotegerin (OR = 0.52, *P* = .046). After further adjustment including VAT, ICAM-1 (OR = 2.67, *P* = .02), adiponectin (OR = 0.57, *P* = .003), and osteoprotegerin (OR = 0.48, *P* = .03) remained significantly associated with fatty liver. We formally tested interactions between HIV and biomarker levels and found a significant negative interaction between HIV and TNFαR2 (*P* = .01) and between HIV and CRP (*P* = .01), supporting our stratified analysis that demonstrated TNFαR2 and CRP were associated with fatty liver only among HIV-uninfected men (Table 3). In sensitivity analysis including HOMA-IR, the associations of the biomarkers with fatty liver were slightly attenuated, but the results were not substantially changed (Supplemental Table 2). The results were also unchanged in another sensitivity analysis excluding HIV-infected participants with detectable viral load (data not shown).

Finally, we evaluated the correlation between biomarker levels and HIV-specific factors. This was notable for an inverse relationship between cumulative dideoxynucleoside use and adiponectin levels (data not shown). In addition, sCD163 was positively correlated with current HIV RNA level and negatively correlated with current CD4 count and HAART use. Adjustment for current CD4, undetectable HIV RNA level, cumulative HAART, and cumulative dideoxynucleoside use in the multivariable logistic regression analysis did not alter our findings for adiponectin (OR = 0.65; 95% confidence interval [CI], 0.44–0.97) or ICAM-1 (OR = 2.79; 95% CI, 1.16–6.67) but did attenuate the association of osteoprotegerin with fatty liver (OR = 0.53; 95% CI, 0.26–1.09).

## DISCUSSION

To our knowledge, this is the largest study to evaluate associations of circulating biomarker levels with fatty liver in a population that included HIV-infected and HIV-uninfected persons. Our first major finding was that higher levels of proinflammatory biomarkers (ICAM-1, CRP, IL-6, and TNFαR2) and lower levels of the anti-inflammatory adiponectin were associated with fatty liver among HIV-uninfected men independent of VAT. Our second major finding was that the association of these marker levels with fatty liver differed by HIV serostatus: among HIV-infected men, ICAM-1 was the only proinflammatory biomarker associated with increased odds of fatty liver, whereas adiponectin and osteoprotegerin were associated with decreased odds.

Our finding that fatty liver was associated with higher TNFαR2 and IL-6 and lower adiponectin levels among HIV-uninfected men is consistent with the current understanding of fatty liver disease pathophysiology. In obesity, visceral adipocyte expansion leads to both increased release of fatty acids from adipose tissue and macrophage infiltration and activation within adipose tissue, resulting in secretion of proinflammatory cytokines [16–20]. Evidence supports a causal association between VAT and fatty liver, mediated by free fatty acids and adipose tissue-derived inflammation, most notably TNFα and IL-6 [3, 21–24]. At the same time, obesity is associated with a decrease in adipose tissue secretion of adiponectin, and adipokine that improves hepatic insulin sensitivity, has several anti-inflammatory properties including production of anti-inflammatory cytokines, and has protective effects in the liver [20, 25–27]. Although our study is cross-sectional and therefore we cannot assess causality, our findings support this model and may extend it because TNFαR2, IL-6, and adiponectin remained associated with fatty liver independent of VAT. One potential explanation for this independent association with fatty liver is that hepatic fat deposition induces expression of regulatory proteins known as hepatokines, which feedback on adipose tissue resulting in further increases in proinflammatory cytokine secretion and decreases in adiponectin secretion [28]. In addition, both TNFα and IL-6 are produced by the liver in response to lipid accumulation [29–31] and suppress adiponectin transcription by adipocytes [32, 33]. Although we are unable to determine the anatomic source of the elevated biomarker levels in our cohort, nor can we determine the direction of our observed associations, our findings underscore the complex interplay and bidirectional relationship between the liver and adipose tissue in fatty liver disease.

It is notable that ICAM-1 and CRP were also associated with fatty liver independent of VAT among HIV-uninfected men. Others have similarly found associations between levels of these markers and central adiposity or fatty liver in HIV-uninfected populations [3, 34, 35]. Intercellular adhesion molecule-1 is a marker of endothelial cell activation and is expressed by lipid-laden hepatocytes in addition to adipose tissue [35]. Similarly, CRP is produced in the liver, and its expression is both induced by IL-6 and increased in patients with nonalcoholic steatohepatitis (NASH) [36, 37]. Our findings are noteworthy because compelling evidence is emerging that fatty liver is independently associated with subclinical and clinical cardiovascular disease, even after adjusting for shared metabolic risk factors [38, 39]. The biomarkers that we found to be independently associated with fatty liver, lower adiponectin, and higher ICAM-1, CRP, IL-6, and TNFαR2 are also predictive of cardiovascular events [40, 41], suggesting a potential explanation for a direct link between fatty liver and cardiovascular events.

A novel finding in our study was the differential association of inflammatory biomarkers and fatty liver by HIV serostatus. Compared with HIV-uninfected men, HIV-infected men had higher TNFαR2 and IL-6 levels, but, interestingly, these markers were not associated with fatty liver in the HIV-infected group, even after excluding those with detectable HIV viral load. One explanation for this is that persistent HIV-induced heightened inflammation despite antiretroviral therapy obscures associations between inflammatory marker levels and fatty liver. More importantly, sCD163, microbial translocation, and Toll-like receptor 4 signaling have been associated with fatty liver progression to NASH and fibrosis [42, 43]. A key outstanding question is whether HIV potentiates these mechanisms in individuals with fatty liver and therefore increases the risk of abnormal histology and disease progression. We were unable to evaluate this in our cohort due to lack of liver histology.

In contrast to our inflammatory marker findings, adiponectin was protective of fatty liver regardless of HIV serostatus, underscoring the importance of the anti-inflammatory properties of adiponectin in fatty liver disease. This finding is supported by increasing evidence linking adiponectin dysregulation to HIV-associated metabolic complications. Circulating adiponectin levels are reduced in patients with HIV-associated lipodystrophy and inversely correlated with insulin resistance [44, 45]. Moreover, certain antiretroviral medications, including elvitegravir, efavirenz, stavudine, ritonavir, and ritonavir-boosted lopinavir, impair adipocyte adiponectin gene expression [46–49]. In contrast, raltegravir does not reduce adiponectin gene expression, and, in fact, switching from stavudine to raltegravir increased gene expression in a group of patients with lipodystrophy [47]. We found an inverse correlation between cumulative dideoxynucleoside use and adiponectin levels, which perhaps explains in part our observed association between this medication class and fatty liver in our cohort. There was not enough raltegravir use in our cohort to determine whether this was protective against fatty liver. Further research is warranted in this area.

Among HIV-infected men, higher ICAM-1 and lower osteoprotegerin levels were independently associated with fatty liver. Interestingly, ICAM-1 levels were not associated with VAT in the HIV-infected men, suggesting that the elevated levels among the HIV-infected men with fatty liver may be a consequence of hepatic expression. Similarly, osteoprotegerin levels were not significantly associated with VAT but were inversely associated with fatty liver in the HIV-infected men. Osteoprotegrin is a decoy receptor for RANKL and TNF-related apoptosis-inducing ligand, is expressed by endothelial cells, vascular smooth muscle cells, and osteoblasts, and has both anti-inflammatory and antiapoptotic effects. Serum osteoprotegerin levels have been inversely associated with insulin resistance and fatty liver [50–52]; thus, its association in our study may be related to the protective effect of osteoprotegrin against metabolic derangements. Further work is needed to understand this association and why osteoprotegrin was associated with fatty liver only in the HIV-infected men.

There are several limitations to our study. First, our definition of fatty liver relied on noncontrast CT scan, which is not sensitive to detect mild fatty liver and has not been validated as a measure of fatty liver in HIV-infected populations. The prevalence of fatty liver in our cohort (15%) was similar to that of another US-based cohort, which used noncontrast CT to assess fatty liver (17%) [53], but lower than that observed in another cohort of HIV-monoinfected individuals who were referred to a metabolic clinic in Italy (37%) [54]. This suggests that the men in our study are relatively healthy compared with those in other HIV cohorts, which may impact the applicability of our findings. Second, the cross-sectional study design impedes our ability to make inferences about the causal pathways associating the biomarkers and our outcomes. Third, in the absence of histology, we cannot differentiate between simple steatosis and steatohepatitis, nor can we assess whether the elevated inflammatory biomarker levels are upregulated in the liver versus elsewhere. Finally, our study included only men, and therefore the findings may not be generalizable to women. This is important because others have found sex differences in the associations of circulating biomarkers and VAT [55]. However, there are also many strengths of our study, including the large sample size, the inclusion of appropriate HIV-uninfected controls, and direct measures of visceral adiposity.

## CONCLUSIONS

In summary, elevated levels of multiple proinflammatory biomarkers, independent of VAT area, were associated with an increased risk of fatty liver in HIV-uninfected men but not in HIV-infected men despite the latter group’s overall heightened proinflammatory state. In contrast, adiponectin was associated with a lower risk of fatty liver regardless of HIV serostatus, highlighting the potential importance of the anti-inflammatory state in protecting against fatty liver. Furthermore, our findings suggest a possible mechanistic link between fatty liver and cardiovascular disease potentially mediated by the alterations in biomarker levels that we observed and support the need for prospective studies to assess these relationships.

## Supplementary Data

Supplementary materials are available at *Open Forum Infectious Diseases* online. Consisting of data provided by the authors to benefit the reader, the posted materials are not copyedited and are the sole responsibility of the authors, so questions or comments should be addressed to the corresponding author.

## Supplementary Material

ofx153_suppl_Supplementary_Data
