# Supplementary material for: The Association of Inflammatory Markers With Nonalcoholic Fatty Liver Disease Differs by Human Immunodeficiency Virus Serostatus
Source: Open Forum Infect Dis. 2017 Jul 23;4(3):ofx153. doi: 10.1093/ofid/ofx153 (PMC5601080; doi:10.1093/ofid/ofx153)
Supplement: ofx153_suppl_Supplementary_Data [file ofx153_suppl_supplementary_data.pdf]

SUPPLEMENTAL DATA

Supplementary Methods

Interassay coefficient of variation ranges for biomarkers: 5.26% to 10.79% for adiponectin, 5.94% to 6.83% for leptin, 4.5% to 5.2% for CRP, 4.14% to 5.1% for cystatin C, 4% to 17% for d-dimer, 1.03% to 6.69% for fibrinogen, 3.99% to 5.22% for sCD14, 1.71% to 5.8% for sCD163, 3.23% to 4.06% for MCP-1, 3.3% to 7% for ICAM-1, 7% to 12% for IL-6, 10.41% to 12.45% for RANKL, 8.74% to 13.53% for osteoprotegerin, 4.62% to 10.8% for sTNFαR1, and 4.23% to 7.93% for sTNFαR2.

Supplemental Figure: Flow-chart showing selection of study population

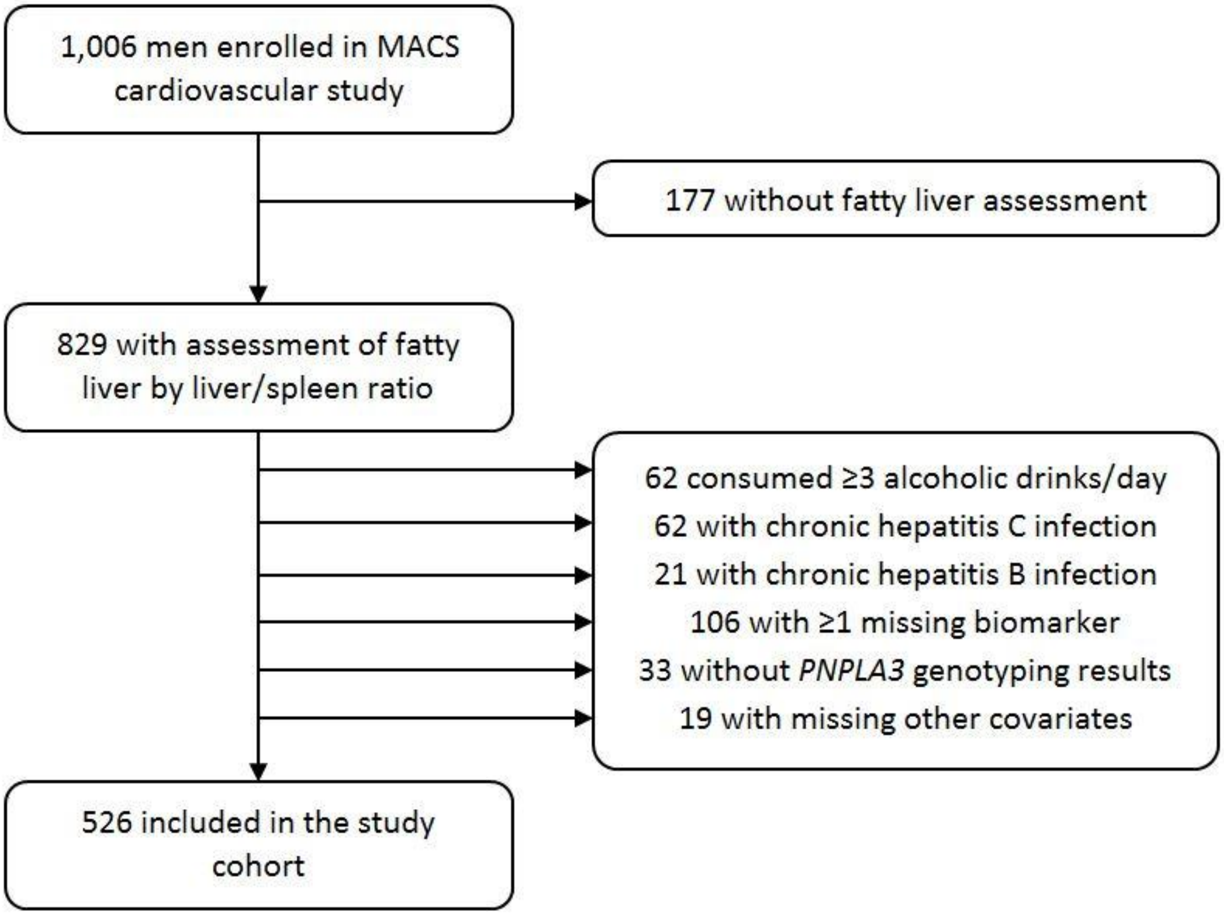

Supplemental Table 1. Association of biomarkers with visceral adipose tissue (VAT) by HIV serostatus

	HIV+ (N=329)		HIV- (N=197)	
	Difference in VAT (95% CI)*	p-value	Difference in VAT (95% CI)*	p-value
ICAM-1†	-1.1 (-14.0, 11.8)	0.87	**30.7 (6.7, 54.7)**	**0.01**
Adiponectin	**-27.4 (-36.5, -18.3)**	**<0.001**	**-28.9 (-43.3, -14.5)**	**<0.001**
CRP†	**12.2 (5.9, 18.4)**	**<0.001**	**24.3 (17.1, 31.6)**	**<0.001**
IL-6†	7.5 (-1.6, 16.6)	0.11	**32.1 (18.9, 45.3)**	**<0.001**
MCP1	13.4 (-1.6, 28.3)	0.08	4.8 (-17.8, 27.3)	0.68
TNFαR2	7.8 (-13.4, 29.0)	0.47	**39.5 (7.9, 71.0)**	**0.02**
Osteoprotegerin	-7.8 (-27.5, 11.9)	0.44	0.1 (-30.5, 30.8)	0.99
sCD163	1.8 (-2.2, 5.9)	0.37	**7.4 (1.4, 13.4)**	**0.02**
sCD14	**-18.6 (-32.1, -5.1)**	**0.007**	1.1 (-23.4, 25.6)	0.93
Cystatin C	26.5 (-4.2, 57.2)	0.09	**108.8 (54.0, 163.6)**	**<0.001**
D-dimer	2.4 (-5.3, 10.1)	0.54	**10.4 (0.6, 20.1)**	**0.04**
Fibrinogen	-0.9 (-17.4, 15.6)	0.91	36.5 (-8.5, 81.6)	0.11
IL-18	3.2 (-6.5, 12.9)	0.51	3.5 (-6.4, 13.4)	0.49
RANKL	-1.8 (-7.2, 3.5)	0.51	3.1 (-4.2, 10.3)	0.41
TNFαR1	15.1 (-6.6, 36.8)	0.17	**29.1 (5.0, 53.2)**	**0.02**
Leptin	**37.8 (31.9, 43.7)**	**<0.001**	**45.6 (37.8, 53.4)**	**<0.001**

Bold signifies statistical significance.
*Difference in VAT was calculated using multivariable linear regression models for each 2 times increase in the levels of biomarkers. All models were adjusted for MACS study site, age and race.
†Test for interaction between HIV and biomarker was statistically significant with $p<0.05$
Abbreviations: sCD14, soluble CD14; sCD163, soluble CD163; ICAM-1, intercellular adhesion molecule 1; IL-6, interleukin 6; IL-18, interleukin 18; MCP1, monocyte chemoattractant protein 1; RANKL, receptor activator of nuclear factor kappa B ligand; TNFαR1, tumor necrosis factor α receptor 1; TNFαR2, tumor necrosis factor α receptor 2; VAT, abdominal visceral adipose tissue; CI, confidence interval

Supplemental Table 2. Association of biomarkers with fatty liver by HIV serostatus

		HIV+ (N=329)		HIV- (N=197)		
		Odds ratio for fatty liver (95% CI)*	p-value	Odds ratio for fatty liver (95% CI)*	p-value	p for interaction
sCD14	Model A	0.72 (0.49-1.07)	0.10	0.96 (0.46-1.99)	0.90	0.50
	+ HOMA-IR	0.79 (0.54-1.17)	0.24	0.85 (0.41-1.77)	0.67	0.87
	+ VAT	0.83 (0.54-1.27)	0.40	0.97 (0.41-2.27)	0.94	0.80
	+ HOMA-IR + VAT	0.86 (0.56-1.32)	0.48	0.87 (0.39-1.97)	0.75	0.99
sCD163	Model A	1.13 (0.59-2.18)	0.72	**2.99 (1.30-6.90)**	**0.01**	0.06
	+ HOMA-IR	0.73 (0.35-1.50)	0.39	**2.98 (1.19-7.43)**	**0.02**	**0.01**
	+ VAT	0.87 (0.42-1.82)	0.71	2.32 (0.96-5.61)	0.06	0.07
	+ HOMA-IR + VAT	0.66 (0.30-1.45)	0.30	2.49 (0.99-6.30)	0.054	**0.02**
Fibrinogen	Model A	0.94 (0.58-1.51)	0.79	2.81 (0.64-12.27)	0.17	0.15
	+ HOMA-IR	0.88 (0.54-1.44)	0.61	2.33 (0.51-10.65)	0.28	0.22
	+ VAT	0.94 (0.59-1.50)	0.81	2.08 (0.47-9.08)	0.33	0.27
	+ HOMA-IR + VAT	0.90 (0.56-1.44)	0.67	2.12 (0.45-9.90)	0.34	0.25
TNFαR2	Model A	1.19 (0.57-2.46)	0.64	**9.11 (2.78-29.80)**	**<0.001**	**0.004**
	+ HOMA-IR	1.05 (0.49-2.28)	0.90	**7.90 (2.24-27.82)**	**0.001**	**0.01**
	+ VAT	0.94 (0.42-2.09)	0.87	**6.55 (1.90-22.53)**	**0.003**	**0.01**
	+ HOMA-IR + VAT	0.91 (0.41-2.04)	0.82	**6.61 (1.85-23.67)**	**0.004**	**0.01**
Adiponectin	Model A	**0.53 (0.38-0.74)**	**<0.001**	**0.45 (0.27-0.74)**	**0.002**	0.71
	+ HOMA-IR	**0.61 (0.43-0.88)**	**0.01**	**0.53 (0.31-0.91)**	**0.02**	0.72
	+ VAT	**0.57 (0.40-0.83)**	**0.003**	**0.51 (0.29-0.89)**	**0.02**	0.90
	+ HOMA-IR + VAT	**0.61 (0.42-0.90)**	**0.01**	**0.55 (0.31-0.98)**	**0.04**	0.85
CRP	Model A	1.18 (0.94-1.49)	0.15	**2.02 (1.49-2.74)**	**<0.001**	**0.01**
	+ HOMA-IR	1.16 (0.91-1.47)	0.23	**1.74 (1.27-2.39)**	**0.001**	0.07
	+ VAT	1.00 (0.78-1.29)	0.99	**1.66 (1.21-2.30)**	**0.002**	**0.03**
	+ HOMA-IR + VAT	1.02 (0.79-1.32)	0.88	**1.57 (1.13-2.19)**	**0.01**	0.08
Cystatin C	Model A	2.28 (0.81-6.40)	0.12	4.59 (0.76-27.52)	0.10	0.41
	+ HOMA-IR	1.95 (0.64-6.00)	0.24	1.84 (0.28-12.28)	0.53	0.70
	+ VAT	1.55 (0.47-5.09)	0.47	1.43 (0.21-9.66)	0.72	0.67
	+ HOMA-IR + VAT	1.45 (0.42-4.97)	0.55	1.00 (0.14-7.12)	1.00	0.74
D-dimer	Model A	**1.32 (1.01-1.73)**	**0.046**	1.19 (0.88-1.62)	0.26	0.56
	+ HOMA-IR	1.27 (0.95-1.70)	0.10	1.00 (0.73-1.37)	1.00	0.29
	+ VAT	1.34 (1.00-1.79)	0.05	1.07 (0.77-1.48)	0.69	0.28
	+ HOMA-IR + VAT	1.30 (0.96-1.76)	0.09	0.96 (0.69-1.34)	0.82	0.23
ICAM-1	Model A	**3.21 (1.45-7.12)**	**0.004**	**8.18 (2.74-24.41)**	**<0.001**	0.09
	+ HOMA-IR	**2.48 (1.14-5.39)**	**0.02**	**6.00 (1.89-19.06)**	**0.002**	0.11
	+ VAT	**2.67 (1.15-6.19)**	**0.02**	**5.30 (1.71-16.46)**	**0.004**	0.22
	+ HOMA-IR + VAT	**2.41 (1.06-5.49)**	**0.04**	**4.74 (1.46-15.33)**	**0.01**	0.21
IL-18	Model A	1.03 (0.72-1.46)	0.89	0.96 (0.70-1.31)	0.79	0.75
	+ HOMA-IR	0.93 (0.64-1.34)	0.68	0.97 (0.69-1.36)	0.85	0.80
	+ VAT	0.95 (0.65-1.39)	0.79	0.90 (0.62-1.32)	0.60	0.87
	+ HOMA-IR + VAT	0.90 (0.61-1.33)	0.60	0.93 (0.64-1.36)	0.71	0.85
IL-6	Model A	1.26 (0.92-1.72)	0.14	**2.11 (1.38-3.24)**	**0.001**	0.06
	+ HOMA-IR	1.18 (0.84-1.66)	0.34	**1.86 (1.18-2.93)**	**0.01**	0.13
	+ VAT	1.16 (0.81-1.66)	0.42	**1.67 (1.06-2.64)**	**0.03**	0.19
	+ HOMA-IR + VAT	1.12 (0.77-1.62)	0.56	**1.63 (1.01-2.63)**	**0.04**	0.18
Leptin	Model A	**1.46 (1.12-1.91)**	**0.01**	**1.67 (1.18-2.38)**	**0.004**	0.48
	+ HOMA-IR	1.21 (0.91-1.61)	0.20	1.27 (0.86-1.86)	0.23	0.58
	+ VAT	0.93 (0.67-1.31)	0.70	1.02 (0.65-1.59)	0.94	0.58
	+ HOMA-IR + VAT	0.86 (0.61-1.23)	0.41	0.89 (0.56-1.42)	0.62	0.57
MCP1	Model A	1.87 (0.96-3.65)	0.07	1.32 (0.66-2.67)	0.43	0.35
	+ HOMA-IR	1.70 (0.86-3.36)	0.13	1.38 (0.64-2.97)	0.41	0.60
	+ VAT	1.72 (0.85-3.49)	0.13	1.28 (0.58-2.85)	0.54	0.57
	+ HOMA-IR + VAT	1.64 (0.81-3.32)	0.17	1.33 (0.58-3.04)	0.50	0.71

Osteoprotegerin	Model A	**0.52 (0.27-0.99)**	**0.046**	1.73 (0.63-4.77)	0.29	0.08
	+ HOMA-IR	0.50 (0.25-1.01)	0.053	1.36 (0.48-3.89)	0.56	0.14
	+ VAT	**0.48 (0.24-0.94)**	**0.03**	1.67 (0.59-4.76)	0.34	0.07
	+ HOMA-IR + VAT	**0.48 (0.24-0.97)**	**0.04**	1.40 (0.48-4.09)	0.54	0.13
RANKL	Model A	0.99 (0.82-1.19)	0.90	0.99 (0.79-1.24)	0.94	0.86
	+ HOMA-IR	1.01 (0.82-1.23)	0.96	1.08 (0.85-1.38)	0.53	0.65
	+ VAT	1.02 (0.84-1.24)	0.82	0.97 (0.76-1.23)	0.80	0.82
	+ HOMA-IR + VAT	1.03 (0.84-1.25)	0.79	1.04 (0.81-1.33)	0.77	0.92
TNFαR1	Model A	1.25 (0.58-2.74)	0.57	2.34 (0.91-6.02)	0.08	0.18
	+ HOMA-IR	1.13 (0.50-2.56)	0.78	1.80 (0.70-4.62)	0.22	0.24
	+ VAT	0.94 (0.40-2.20)	0.89	1.64 (0.63-4.29)	0.32	0.17
	+ HOMA-IR + VAT	0.91 (0.38-2.18)	0.84	1.53 (0.58-4.03)	0.39	0.18

Bold signifies statistical significance at p-value<0.05.
*Odds ratios are calculated for each 2 times increase in the levels of biomarkers. Model A was adjusted for MACS study site, age, race and *PNPLA3* genotype. P for interaction tested potential interaction between biomarkers and HIV serostatus.
Abbreviations: sCD14, soluble CD14; sCD163, soluble CD163; ICAM-1, intercellular adhesion molecule 1; IL-6, interleukin 6; IL-18, interleukin 18; MCP1, monocyte chemoattractant protein 1; RANKL, receptor activator of nuclear factor kappa B ligand; TNFαR1, tumor necrosis factor α receptor 1; TNFαR2, tumor necrosis factor α receptor 2; OR, odds ratio; CI, confidence interval; HOMA-IR, homeostatic model assessment of insulin resistance; VAT, abdominal visceral adipose tissue.
